# Diffusion and Persistence of Multidrug Resistant *Salmonella* Typhimurium Strains Phage Type DT120 in Southern Italy

**DOI:** 10.1155/2015/265042

**Published:** 2015-04-28

**Authors:** Danila De Vito, Rosa Monno, Federica Nuccio, Marilisa Legretto, Marta Oliva, Maria Franca Coscia, Anna Maria Dionisi, Carla Calia, Carmen Capolongo, Carlo Pazzani

**Affiliations:** ^1^Department of Basic Medical Sciences Neurosciences and Sense Organs Medical Faculty, University of Bari Piazza G. Cesare Policlinico, 70124 Bari, Italy; ^2^Department of Biology, University of Bari “Aldo Moro”, Via E. Orabona 4, 70125 Bari, Italy; ^3^Department of Infectious, Parasitic and Immune-Mediated Diseases, National Health Institute, 00161 Rome, Italy

## Abstract

Sixty-two multidrug resistant* Salmonella enterica* serovar Typhimurium strains isolated from 255 clinical strains collected in Southern Italy in 2006–2008 were characterised for antimicrobial resistance genes, pulsotype, and phage type. Most strains (83.9%) were resistant to ampicillin, chloramphenicol, streptomycin, sulfamethoxazole, and tetracycline (ACSSuT) encoded in 88.5% by the* Salmonella* genomic island (SGI1) and in 11.5% by the InH-like integron (*bla*_OXA-30_–*aadA1*) and* catA1*,* sul1*, and *tet*(B) genes. STYMXB.0061 (75%) and DT120 (84.6%) were the prevalent pulsotype and phage type identified in these strains, respectively. Five other resistance patterns were found either in single or in a low number of isolates. The pandemic clone DT104 (ACSSuT encoded by SGI1) has been identified in Italy since 1992, while strains DT120 (ACSSuT encoded by SGI1) have never been previously reported in Italy. In Europe, clinical strains DT120 have been reported from sporadic outbreaks linked to the consumption of pork products. However, none of these strains were STYMXB.0061 and SGI1 positive. The prevalent identification and persistence of DT120 isolates would suggest, in Southern Italy, a phage type shifting of the pandemic DT104 clone pulsotype STYMXB.0061. Additionally, these findings raise epidemiological concern about the potential diffusion of these emerging multidrug resistant (SGI linked) DT120 strains.

## 1. Introduction

In recent times, diseases caused by foodborne pathogens have been the object of an increasing number of studies [1]. This is owing to the rates of morbidity and mortality that can be achieved by such diseases and the impact that they have in both public health and social economic costs. Among the different bacteria responsible for foodborne diseases,* Salmonella enterica* represents one of the leading causes of such infections with* S.* Enteritidis and* S.* Typhimurium being the most common serovars isolated from humans in Europe and the USA [2]. However, while in Europe* S.* Enteritidis still remains the predominant serovar from clinical cases, in Italy it is the serovar* S.* Typhimurium which is prevalent [3].

In the past decades, the emergence and spread of* Salmonella* multidrug resistant (MDR) strains have also contributed to increasing the impact that these pathogens have had in public health. The insurgence of MDR has generally been linked to acquisition of resistance genes located on plasmids, transposons, genomic islands, and integrons. For example, the multidrug resistance exhibited by the pandemic* S.* Typhimurium definitive phage type 104, (DT104), is due to resistance genes located within a chromosomal genomic island termed SGI1 composed of a 43 kbp region in which lies a 13 kbp gene cluster of* aadA2*,* floR*,* tet*(G), *bla*_PSE-1_, and* sul1* encoding resistance for streptomycin, chloramphenicol, tetracycline, ampicillin, and sulfamethoxazole, respectively [4].

The constant increase of antimicrobial resistance is a global scale problem and it raises concern on the choice of antimicrobials available for the treatment of bacterial infections, particularly for those pathogens of major social relevance. It is thus necessary to implement measures that may contribute to better assessing the extent of resistance-gene diffusion among these pathogens. Both monitoring and characterisation of the antimicrobial susceptibility are undoubtedly valuable investigation systems that fulfill the purpose of such measures. Antimicrobial resistance is usually acquired by point mutations in the bacterial genome or by horizontal transfer of genetic elements harbouring resistance genes. Additionally, gene capture and expression elements called integrons have recently been shown to play an important role in acquisition and dissemination of resistance genes. Among the different classes of integrons, class 1 is the most detected and found strongly associated with capture of antimicrobial resistance genes. Class 1 integrons are then recognised as major contributors to the problem of multidrug-resistant pathogens [5]. In* S. enterica*, class 1 integrons are common and greatly contribute to the extent of antimicrobial resistance in this species [6].

If genetic characterization of antimicrobial resistances was combined with molecular strain typing, it would greatly benefit knowledge on the spread of resistance genes among the clonal groups of* Salmonella* strains principally identified in the clinical field. Phage typing and pulsed-field gel electrophoresis (PFGE) have become universally recognized as the reference methods for* Salmonella* strain subtyping.

In Italy, until 2006, the most common phage types identified among MDR clinical* S.* Typhimurium strains were DT104, U302, and DT7 [3]. The first was strongly associated with isolates pulsotype STYMXB.0061 resistant to ampicillin, chloramphenicol, streptomycin, sulfamethoxazole, and tetracycline, while strains U302 and DT7 were pulsotype STYMXB.0079 and resistant to ampicillin, streptomycin, sulfamethoxazole, and tetracycline. However, in Southern Italy (where* Salmonella* infections are endemic), data on resistance genes and clonal relationship among the MDR strains isolated from clinical cases is poor [7]. In this study, MDR* S.* Typhimurium strains isolated from several hospitals, from 2006 to 2008, were phage typed and characterised by pulsed field gel electrophoresis (PFGE). The genetic basis of the antimicrobial resistance was also established.

## 2. Materials and Methods

### 2.1. Bacterial Isolates and Antimicrobial Susceptibility Testing

From January 2006 to December 2008, four hundred and seventy-seven* Salmonella* strains were isolated from clinical cases which occurred in Southern Italy. Two hundred and fifty-five were* S. enterica* serovar Typhimurium. Strains were isolated from six different hospitals scattered among the Italian neighbouring regions of Apulia and Basilicata. Biochemical identification of* Salmonella* was confirmed by an automated system (Microscan, dade Behring, Milan, Italy) and serotyping was performed by agglutination tests with specific antisera O and H antigens (Bio-Rad Laboratories, Italy). Classification was performed according to the Kauffmann-White scheme [8].

All isolates were included in the database of the Enter-net Surveillance Network at the Institute of Hygiene of the University of Bari (Italy) coordinated by the Istituto Superiore di Sanità, Rome (Italy). There were sixty-two bacteria that were resistant to at least one agent in three or more of the antimicrobial categories tested (namely, aminoglycosides, folate pathway inhibitors, penicillins, phenicols, and tetracyclines) and so defined as MDR [11].

Antimicrobial susceptibility testing was carried out by the agar disk diffusion test on Mueller-Hinton agar (Oxoid, Milan, Italy), following the Clinical and Laboratory Standards Institute (CLSI) guidelines [12]. The antimicrobial disks were ampicillin (A; 10 *μ*g), chloramphenicol (C; 30 *μ*g), kanamycin (K; 30 *μ*g), streptomycin (S; 10 *μ*g), sulfamethoxazole (Su; 25 *μ*g), tetracycline (T; 30 *μ*g), and trimethoprim (Tp 5 *μ*g).* E. coli* ATCC 25922 was used as a quality control strain.

### 2.2. PCR Detection of SGI, Class 1 Integrons, and Antimicrobial Resistance Genes

Primers used to detect SGI1, class 1 integrons, and the antimicrobial resistance genes* aadA1, aadA2*, and* strAB* (encoding resistance for streptomycin; category aminoglycosides), *bla*_PSE-1_ and *bla*_TEM_ (encoding resistance for ampicillin; category penicillins),* floR* (encoding resistance for chloramphenicol; category phenicols),* sul1* and* sul2* (encoding resistance for sulfamethoxazole; category folate pathway inhibitors), and* tet*(B),* tet*(C), and* tet*(G) (encoding resistance for tetracycline; category tetracyclines) were as reported previously [9, 10]. Primers to detect the antimicrobial resistance genes *bla*_OXA-30_ (encoding resistance for ampicillin),* catA1* (encoding resistance for chloramphenicol),* dfrA1* and* dfrA12* (encoding resistance for trimethoprim; category folate pathway inhibitors), and* tet*(A) and* tet*(D) (encoding resistance for tetracycline) were designated in this study. All primers are listed in Table 1. The primers employed in the current study were based on sequences available in GenBank and designed by using the Primer3 (version 4.0.0) software (http://primer3.wi.mit.edu). Genomic DNA was extracted as previously described [13]. PCRs were performed in a total volume of 25 *μ*L containing 50 to 100 ng of total DNA, 1X PCR buffer (10 mM Tris-HCl, 50 mM KCl, and 1.5 mM MgCl_2_; pH8.3), 200 *μ*M of each deoxynucleoside triphosphate (dNTP), 20 *μ*M of each primer, and 1 U* Taq* polymerase (Takara Bio Inc., Otsu, Shiga, Japan).

Antimicrobial gene cassettes integrated into class 1 integrons were amplified with primers 5CS-F and 3CS-R and cloned into pGEM-T Easy vector (Promega, Milan, Italy) in accordance with the manufacturer's instructions.* E. coli* JM109 was used as a recipient strain. The cloned products were purified using the Pure Yield Plasmid Miniprep System (Promega) and sequenced by the Big Dye Terminator method (BMR Genomics, Padova, Italy). The resulting DNA sequences were analysed for similarity by using the BLAST program available on the NCBI BLAST homepage (http://www.ncbi.nlm.nih.gov/BLAST).

### 2.3. PFGE and Phage Typing

Genomic restriction was performed according to the standardized PulseNet* Salmonella* protocol [14]. Agarose-embedded DNA was digested with 40 U of* Xba*I for 3 h at 37°C. The restriction fragments were separated by electrophoresis in Tris-borate-EDTA (44.5 mM Tris-borate, 1 mM EDTA; pH 8.0) at 14°C for 20 h using a CHEF-DR III (Bio-Rad, Milan, Italy). Electrophoresis conditions were as follows: 6 V/cm, angle of 120°C, for 20 h with pulse times of 2.2 to 63.8 s. The* Salmonella* Braenderup H9812 strain was used as a molecular standard. The PFGE agarose gels were stained with ethidium bromide (40 *μ*g/mL) and the DNA band images were acquired by the Gel Doc-It photo documentation system (Gel Doc-It photo documentation system, UVP, Upland, CA, USA).

**Figure 1:**
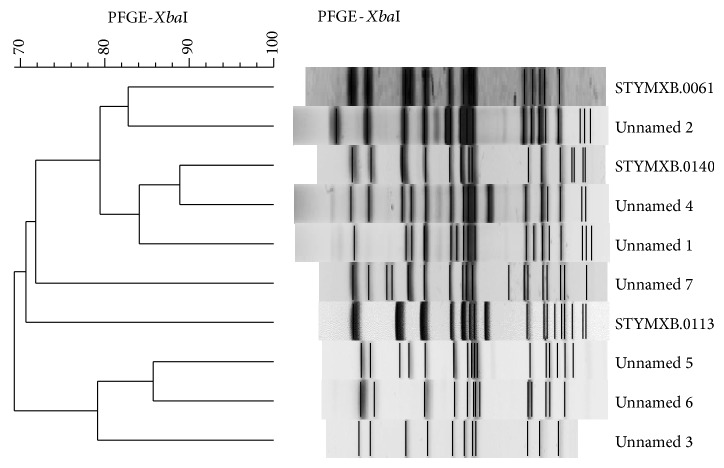
PFGE profiles and cluster analysis of* Salmonella enterica* serotype typhimurium strains analysed in this study. All representative variants of single PFGE patterns are included.

PFGE profiles in digital tagged image file format (TIFF files) were analyzed with the BioNumerics software package v.6.6 (Applied Maths, Sint-Martens-Latem, Belgium) [15]. DNA profiles differing in one or more DNA fragments were considered as distinct patterns. Strains with a coefficient of similarity ≥90% were classified as genetically closely related. All PFGE profiles were compared with those included in the PulseNet-Europe international database and named with a six letter code followed by a four digit numerical identifier, for example, STYMXB.0006. Dendrogram and cluster analysis were performed using algorithms available within the BioNumerics software package v.6.6 (Figure 1). Percent similarity between different chromosomal fingerprints was scored by the Dice coefficient. The unweighted pair group method with arithmetic means (UPGMA), with a 1.00% tolerance limit and 1.00% optimisation, was used to obtain the dendrogram. DNA profiles differing by one or more DNA fragments were considered as distinct patterns. Phage typing was performed according to the standard procedure [16].

## 3. Results

### 3.1. Antimicrobial Susceptibility, Pulsotype, and Phage Typing

Six distinct resistance patterns were identified within the 62 MDR* S.* typhimurium strains analysed in this study (Table 2). The resistance pattern to ampicillin, chloramphenicol, streptomycin, sulfamethoxazole, tetracycline, and trimethoprim (termed resistance pattern ACSSuT) was that prevalent (83.9%), being identified in 52 strains. All others resistance patterns were identified either in single (ASSuT, ASSuTp, and AKSSuT) or in a low number of isolates (ASuTTp and ACSSuTTp).

Strains were characterised by genomic* Xba*I restriction patterns and resolved through a pulsed field gel electrophoresis and phage typing. PFGE profiles were compared with those included in the PulseNet-Europe international database and specific pulsotypes were assigned when the coefficient of similarity had a value ≥90%. Among the 52 strains with resistance pattern ACSSuT, 39 were pulsotype STYMXB.0061 with phage types distributed as follows: DT120 (84.6%), U302 (13.5%), and RDNC (1.9%). The remaining 13 strains with resistance pattern ACSSuT were pulsotypes STYMXB.0140 (4) or unnamed (9), and all of them were classified as phage type DT120. Five strains resistance pattern ASuTTp were pulsotype STYMXB.0113: two strains were classified DT35 and three DT193. Two isolates exhibiting the resistance pattern ACSSuTTp and the single isolates with resistance patterns ASSuT, ASSuTp, and AKSSuT were pulsotype unnamed and phage types DT120, DT7var, DT1, and U302, respectively.

### 3.2. Resistance Genes Associated with SGI1

Identification of SGI1 and associated resistance genes was performed by PCR. Among the 52 strains with resistance pattern ACSSuT, 46 were positive for the presence of SGI1. Resistance genes (*aadA2*,* floR*,* tet*(G), *bla*_PSE-1_, and* sul1*) usually harboured by SGI1 were identified and their genetic organization proved indistinguishable from that reported for SGI1 [4]. SGI1 and associated resistance genes were also identified in strains with resistance pattern ACSSuTTp and found identical to that identified in strains with resistance pattern ACSSuT. Strains with resistance patterns ASuTTp, AKSSuT, ASSuT, and ASSuTp were all negative for the presence of SGI1.

### 3.3. Resistance Genes and Class 1 Integrons

Class 1 integrons were identified in all strains with resistance patterns ACSSuT and ACSSuTTp. Two amplicons of 1,009 bp and 1,197 bp were obtained among the 46 strains with resistance pattern ACSSuT and SGI1 positive. The nucleotide sequence of the gene cassettes was determined and two open reading frames (ORFs) of 852 and 987 bp were detected and found homologous to* aadA2* and *bla*_PSE-1_, respectively. PCR analysis was further extended for detection of* sul1* and its linkage to class 1 integron. The* sul1* gene was found associated only with the class 1 integron harbouring the *bla*_PSE-1_ gene cassette. These data further confirmed the presence of SGI1. An amplicon of 2,013 bp was obtained from the remaining 6 strains with resistance pattern ACSSuT (SGI1 negative). Two ORFs of 813 and 987 bp, organised as a gene cassette array, were identified and found homologous to *bla*_OXA-30_ and* aadA1*. The presence of both *bla*_OXA-30_ and* aadA1*, in these 6 isolates, was also confirmed by PCR and* sul1* was found associated with the gene cassette array. Detection of* catA1* and* tet*(B) completed the analysis of the resistance genes in these 6 isolates.

The strains with resistance pattern ACSSuTTp additional to SGI1, indistinguishable from that identified in strains with resistance pattern ACSSuT, harboured another class1 integron element. The gene cassette amplicon (1,534 bp) was sequenced and two ORFs of 474 and 789 bp were identified. The ORFs were found homologous to* dfrA1* and* aadA1*, respectively.

In strains with resistance pattern ASuTTp, the identified resistance genes were *bla*_TEM_,* sul2*,* tet*(B), and* dfrA12*. In the single strains with resistance patterns AKSSuT, ASSuT, and ASSuTp, the resistance genes were *bla*_TEM_,* sul2*,* strAB*,* tet*(B); *bla*_TEM_,* sul2*,* strAB*,* tet*(A); and *bla*_TEM_,* sul2*,* dfrA12*, respectively. The genetic basis for resistances to kanamycin (resistance pattern AKSSuT) and streptomycin (resistance pattern ASSuTp) was not identified.

## 4. Discussion

*Salmonella* infections still remain a major public health concern in many countries with an estimated incidence of 15.1 cases per 100,000 persons in the United States and 42.2 cases per 100,000 persons in the EU [17]. In Italy, the average annual incidence is 14 cases per 100,000 persons [18]. However, since* Salmonella* infections are generally based on the identification of* Salmonella* strains from stool culture, data officially reported are likely to represent only a fraction of infections which really occur. The difference between reported cases and those probably occurring has recently been investigated by a study that assessed the seroincidence of* Salmonella* infections in eight European countries, Italy included [18]. The study demonstrated the misleading data on the incidence of* Salmonella* infections based only on numbers of stool culture-confirmed cases. Hence, the need to integrate epidemiological data with those gained by novel methods of investigation such as the assessment of cases by seroincidence.

*Salmonella* is also the leading cause of hospitalization from foodborne pathogens and the increase of antimicrobial resistance often represents a problem for the choice of a proper antimicrobial therapy (if required). A clear example is provided by the insurgence in the mid-1980s (in the United Kingdom) of the MDR* S.* Typhimurium phage type 104, resistance pattern ACSSuT encoded by SGI1, and its subsequent diffusion. Shortly after its emergence, detection of the MDR* S.* Typhimurium DT140 constantly increased among 29 countries which took part in a survey conducted from 1992 to 2001 [19]. In the following years, this clone became virtually pandemic.

In Italy, a survey conducted from 1992 to 2000 highlighted the presence of this pandemic clone from 1992 [20]. Detection of the MDR* S.* Typhimurium DT104, resistance pattern ACSSuT, remained constant from 2002 to 2004 accounting for 20% of the* S.* Typhimurium strains of clinical origin [21].

In this study, we report the characterisation of 62 MDR* S.* Typhimurium strains isolated from clinical cases which occurred in Southern Italy from 2004 to 2006. The incidence of* S.* Typhimurium was of 53.5% (255 strains out of the 477), data that confirmed the prevalent detection of this serovar in Italy. Multidrug resistance was detected in 24.3% of the* S.* Typhimurium strains. STYMXB.0061 was the prevalent (62.9%) identified pulsotype. This datum is in agreement with that reported by other studies STYMXB.0061 being one of the most common profiles identified in* S.* Typhimurium [22] and in Italy reported as the second PFGE profile detected in human isolates from 2003 to 2006 [23]. STYMXB.0061 is strongly associated with the phage type 104 and this relationship is documented by a number of studies on* S.* Typhimurium strains isolated from both human and animal sources [10, 22, 23]. SGI1, first detected in* S.* Typhimurium strains phage type 104, has also been identified in strains with phage types related to DT104 such as DT120, U302, or DT12 [24]. Strains DT120 or DT12 with resistance pattern ACSSuT exhibited PFGE profiles identical to those shown by isolates DT104 with resistance pattern ACSSuT and distinct from profiles exhibited by strains of the same phage types [25]. Such a feature has been supposed due to a change in phage susceptibility in a small proportion of* S.* Typhimurium strains phage type 104 with resistance pattern ACSSuT rather than to horizontal gene transfer of resistance determinants. Additionally, phage typing has widely been used for strain characterisation, and in surveillance and outbreaks investigation [26]. Interestingly, in our survey, none of the isolates STYMXB.0061 with resistance pattern ACSSuT was DT104. Thirty-one (79.5%) were phage type DT120 and seven (15.2%) phage type U302. The prevalent identification of phage type DT120 would suggest, at least in Southern Italy, a phage type shifting of the* S.* Typhimurium strain DT104 (resistance pattern ACSSuT, pulsotype STYMXB.0061) of clinical origin. This possibility is also supported by the virtual absence of phage type DT120 detection in* S.* Typhimurium strains of human and non-human sources isolated in Italy at least until 2000 [27, 28]. In a subsequent study performed in Italy (2002–2004) on* S.* Typhimurium strains isolated from human and animal sources, DT120 was rarely detected among the 367 identified MDR strains (resistance pattern ACSSuT). Only 7 isolates were DT120 while 196 (72.3%) were DT104. Additionally, for all of these 7 strains, neither source of isolation nor antimicrobial resistance genes were specified [3].

MDR* S.* Typhimurium DT120 strains (e.g. resistant to ASSuT, encoded by *bla*_TEM_,* strAB*,* sul2* and* tet*(B)) have recently emerged in European countries, with pigs being the likely reservoir of infection [29]. Indeed, in a survey on the prevalence of* Salmonella* in slaughtered pigs, conducted in the EU from 2006 to 2007, the serovars Typhimurium and Derby were those most frequently identified [30].

The potential impact of* S.* Typhimurium DT120 in public health has recently been highlighted by clinical outbreaks which occurred in Denmark in 2008 and the UK in 2011. Infections linked to the consumption of pork or pork products [31–34]. Moreover, in Greece, DT120 is associated with the most frequently occurring clones in human and pigs (2006–2011) [33]. However, none of these DT120 isolates was STYMXB.0061 (STYMXB.0083 was the pulsotype detected from the outbreak in UK, while STYMXB.0010 was that identified in Denmark and Greece) and, above all, the antimicrobial resistance patterns (mainly ASSuT and ACSSuTTp) were not linked to the presence of SGI1.

In Italy, the phage type DT120 has commonly been detected from finishing pigs in slaughterhouses [35]. However, these data come from slaughterhouses located in Northern Italy while data from Southern Italy, to the best of our knowledge, are still unavailable. Additionally, none of the* S.* Typhimurium DT120 strains isolated from slaughterhouses in Northern Italy was resistance pattern ACSSuT and no data was available on their pulsotype. It would be interesting to investigate the molecular and phenotypic features of* S.* Typhimurium strains isolated from swine samples in Southern Italy. This might help determine possible epidemiological routes that have contributed to the spread of* S.* Typhimurium strains phage type DT120 that we detected from the clinical isolates.

The integrons* dfrA1*-*aadA1* and *bla*_OXA-30_-*aadA1* have been identified in* S.* Typhimurium strains isolated from different geographic areas [6]. The identification of distinct groups of integrons together with phage typing and molecular strain characterisation represents valuable markers to assess possible clonal expansion. Interestingly, the above types of integrons were all identified in MDR strains DT120 with different pulsotype (STYMXB.0113 or Unnamed) highlighting the presence of distinct DT120 clones among clinical cases in Southern Italy.

## 5. Conclusions

In this study, we first described the extent of the antimicrobial resistance genes and associated genetic elements among clinical MDR* S.* Typhimurium strains isolated from an Italian area where Salmonella infections are endemic. Data presented suggest, in this endemic area, a phage type shifting of the pandemic clone DT104 (resistance pattern ACSSuT) to DT120. Such a shifting, to the best of our knowledge, has occurred recently since no clinical* S.* Typhimurium strains DT120 (resistance pattern ACSSuT encoded by SGI encoded) was not reported until 2004. The extent and persistence of these findings raise concerns in public health about the possible diffusion of these emerging DT120 clones.

## Figures and Tables

**Table 1:** Primers used for PCR amplification of resistance genes.

Primer	5′-3′ sequence	Gene target	Amplicon size (bp)	TA	Reference
aadA1-F	TTTGATCAACGACCTTTTGGAAAC	*aadA1 *and *aadA2 *	294	58°C	[9]
aadA-R	GGACAACGTAAGCACTACATTTCG				

blaoxa30-F	ATTATCTACAGCAGCGCCAGTGCATC	*bla*_ OXA-30_	716	63°C	This study
blaoxa30-R	TTCGACCCCAAGTTTCCTGTAAGTGC				

blaPSE-1-F	GGATTACAATGGCAATCAGCGCTTCC	*bla*_ PSE-1_	658	65°C	[10]
blaPSE-1-R	AATCGCATCATTTCGCTCTGCCATTG				

blaTEM1-F	TGAAGATCAGTTGGGTGCACGAGTGG	*bla*_ TEM-1_	700	63°C	[9]
blaTEM1-R	AGTTGCCTGACTCCCCGTCGTGTAGA				

catA1-F	CACCGTTGATATATCCCAATGGCATCGT	*catA1 *	582	62°C	This study
catA1-R	CTGCCGACATGGAAGCCATCACAAAC				

dfrA1-F	TATTCCATGGAGTGCCAAAGGTGAAC	*dfrA1 *	375	63°C	This study
dfrA1-R	GAGGCGAAGTCTTGGGTAAAAACTGG				

dfrA12-F	CAGTACGCATTTATCTCGTTGCTGCG	*dfrA12 *	417	63°C	This study
dfrA12-R	CAAGCTCGAATTCTGTTTCGTTGAGC				

floR-F	CTCCTTTCGACATCCTCGCTTCACTG	*floR *	636	64°C	[10]
floR-R	AGAAGACGAAGAAGGTGCCCATACCG				

sul1-F	TCGGCATTCTGAATCTCACCGAGGAC	*sul1 *	786	64°C	[10]
sul1-R	AAATTTCGCGAGGGTTTCCGAGAAGG				

sul2-F	GACAGTTTCTCCGATGGAGGCCGGTA	*sul2 *	700	64°C	[10]
sul2-R	GTGTGCGGATGAAGTCAGCTCCACCT				

strA-F	TGGTTGCCTGTCAGAGGCGGAGAATC	*strAB *	1466	63°C	[9]
strB-R	ATCGACGTCCAGCGCACGAGAGAATG				

tetA-F	CCTGATCGTAATTCTGAGCACTGTCG	*tet*(A)	1089	63°C	This study
tetA-R	CACCCGTTCCACGTTGTTATAGAAGC				

tetB-F	CAGGTTATCTTTGCTCCTTGGCTTGG	*tet*(B)	1014	63°C	[9]
tetB-R	TTGAGGGGTTAACATGAAGGTCATCG				

tetC-F	GGATATCGTCCATTCCGACAGCATCG	*tet*(C)	745	63°C	[9]
tetC-R	GATAATGGCCTGCTTCTCGCCGAAAC				

tetD-F	ACTGTCCAATGTGCTGTGGATGTTGT	*tet(D) *	747	63°C	This study
tetD-R	CTGACCAGCACACCCTGTAGTTTTCC				

tetG-F	GAGCCGCAGTCGATTACACGATTATG	*tet*(G)	680	64°C	[10]
tetG-R	CAACAGAATCGGGAACACCATCCATC				

intI1-F	CGAACCGAACAGGCTTATGTCCACTG	*intI1 *	838	63°C	[10]
intI1-R	CATCGTCGTAGAGACGTCGGAATGG				

5CS-F	GCCTCGGGCATCCAAGCAGCAAGC	5′CS *attI*1 end 3′CS	variable	65°C	This study
3CS-R	CTTGACCTGATAGTTTGGCTGTGAGCAA				

U7-L12	ACACCTTGAGCAGGGCAAAG	*tdhF *	500	60°C	[4]
LJ-R1	AGTTCTAAAGGTTCGTAGTCG	*int *			

104-RJ	TGACGAGCTGAAGCGAATTG	S044	515	60°C	[4]
C9-L	AGCAAGTGTGCGTAATTTGG	*int2 *			

**Table 2:** Antimicrobial susceptibility, phage types, pulsotypes, PCR detection of SGI1, class 1 integrons, and resistance genes in *S*. Typhimurium strains isolated in Southern Italy in 2006–2008.

PulseNet Europe nomenclature	Phage type (no. of strains)	Year (no. of strains)	Resistance pattern^a^		Class 1 integrons		SGI1	
				*intI1 *	Gene cassette(s)	Resistance gene(s)	Left junction	Right junction
STYMXB.0061	DT120 (31)	2006 (9); 2007 (13); 2008 (9)	ACSSuT	+	*aadA2; bla*_ PSE-1_	*floR; sul1; tet(G) *	+	+
	U302 (7)	2006 (1); 2008 (4) 2008 (2)	′′	+	′′	′′	+	+
			′′	+	′′	′′	+	−
	RDNC (1)	2006 (1)	′′	+	′′	′′	+	+

STYMXB.0113	ST193 (3)	2006	ASuTTp	−	−	*bla*_ TEM_ *; sul2; tet(A); dfrA12 *	−	−
	DT35 (2)^b^	′′	′′	−	−	*bla*_ TEM_ *; sul2; tet(A) *	−	−

STYMXB.0140	DT120 (4)	2007 (4)	ACSSuT	+	*bla*_ OXA-30_-*aadA1 *	*catA1*; *sul1*; *tet(B) *	−	−

Unnamed 1	DT120 (9)	2006 (2); 2007 (3)	ACSSuT	+	*aadA2*;*bla*_PSE-1_	*floR*; *sul1*; *tet(G) *	+	+
Unnamed 2	′′	2008 (2)	′′	+	′′	′′	+	+
Unnamed 3	′′	2008 (2)	′′	+	*bla*_ OXA-30_-*aadA1 *	*catA1*; *sul1*; *tet(B) *	−	−
Unnamed 4	′′	2007 (2)	ACSSuTTp	+	*aadA2; bla*_ PSE-1_ *dfrA1*-*aadA1 *	*floR*; *sul1*; *tet(G) *	+	+
Unnamed 5	DT7var^c^	2007 (1)	AKSSuT	−	−	*bla*_ TEM_;* sul2*;* strAB*;* tet(B) *	−	−
Unnamed 6	DT1	2008 (1)	ASSuT	−	−	*bla*_ TEM_;* sul2*; *strAB*; *tet(A) *	−	−
Unnamed 7	U302^d^	2008 (1)	ASSuTp	−	−	*bla*_ TEM_;* sul2*; *dfrA12 *	−	−

^a^A, ampicillin; C, chloramphenicol; K, kanamycin; S streptomycin; Su, sulfamethoxazole; T, tetracycline; and Tp, trimethoprim.

^b^The resistance to Tp was not identified.

^c^The resistance to K was not identified.

^d^The resistance to S and Tp was not identified.

RDNC: the reaction pattern is not conforming to a recognised phage type.

Key: +, positive; −, negative.

## References

[1] Zhao X., Lin C.-W., Wang J., Oh D. H. (2014). Advances in rapid detection methods for foodborne pathogens. *Journal of Microbiology and Biotechnology*.

[2] Weill F.-X., Guesnier F., Guibert V. (2006). Multidrug resistance in *Salmonella enterica* serotype typhimurium from humans in France (1993 to 2003). *Journal of Clinical Microbiology*.

[3] Graziani C., Busani L., Dionisi A. M. (2008). Antimicrobial resistance in *Salmonella enterica* serovar Typhimurium from human and animal sources in Italy. *Veterinary Microbiology*.

[4] Boyd D., Peters G. A., Cloeckaert A. (2001). Complete nucleotide sequence of a 43-kilobase genomic island associated with the multidrug resistance region of *Salmonella enterica* serovar typhimurium DT104 and its identification in phage type DT120 and serovar agona. *Journal of Bacteriology*.

[5] Hall R. M., Collis C. M. (1998). Antibiotic resistance in gram-negative bacteria: the role of gene cassettes and integrons. *Drug Resistance Updates*.

[6] Krauland M. G., Marsh J. W., Paterson D. L., Harrison L. H. (2009). Integron-mediated multidrug resistance in a global collection of nontyphoidal Salmonella enterica isolates. *Emerging Infectious Diseases*.

[7] Monno R., Rizzo C., de Vito D. (2007). Prevalence, antimicrobial resistance, and extended-spectrum *β*-lactamases characterization of *Salmonella* isolates in Apulia, southern Italy (2001–2005). *Microbial Drug Resistance*.

[8] Popoff M. Y., Bockemühl J., Gheesling L. L. (2004). Supplement 2002 (no. 46) to the Kauffmann-White scheme. *Research in Microbiology*.

[9] Camarda A., Pugliese N., Pupillo A. (2013). Resistance genes, phage types and pulsed field gel electrophoresis pulsotypes in *Salmonella enterica* strains from laying hen farms in southern Italy. *International Journal of Environmental Research and Public Health*.

[10] Camarda A., Pupillo A., Pugliese N. (2013). Phenotypic and genetic traits of *Salmonella enterica* subsp. serovar Typhimurium strains causing salmonellosis foci in rabbit farms from Southern Italy in 1999–2003. *Research in Veterinary Science*.

[11] Magiorakos A.-P., Srinivasan A., Carey R. B. (2012). Multidrug-resistant, extensively drug-resistant and pandrug-resistant bacteria: an international expert proposal for interim standard definitions for acquired resistance. *Clinical Microbiology and Infection*.

[12] Clinical and Laboratory Standards Institute (2006). *Performance Standard for Antimicrobial Disk Susceptibility Tests; Approved Standard M2-A9*.

[13] Pugliese N., Maimone F., Scrascia M., Materu S. F., Pazzani C. (2009). SXT-related integrating conjugative element and IncC plasmids in Vibrio cholerae O1 strains in Eastern Africa. *The Journal of Antimicrobial Chemotherapy*.

[14] Ribot E. M., Fair M. A., Gautom R. (2006). Standardization of pulsed-field gel electrophoresis protocols for the subtyping of *Escherichia coli* O157:H7, *Salmonella*, and *Shigella* for PulseNet. *Foodborne Pathogens and Disease*.

[15] Busani L., Graziani C., Battisti A. (2004). Antibiotic resistance in *Salmonella enterica* serotypes Typhimurium, Enteritidis and Infantis from human infections, foodstuffs and farm animals in Italy. *Epidemiology and Infection*.

[16] Ward L. R., de Sa J. D., Rowe B. (1987). A phage-typing scheme for *Salmonella enteritidis*. *Epidemiology and Infection*.

[17] Alcaine S. D., Warnick L. D., Wiedmann M. (2007). Antimicrobial resistance in nontyphoidal *Salmonella*. *Journal of Food Protection*.

[18] Falkenhorst G., Simonsen J., Ceper T. H. (2012). Serological cross-sectional studies on salmonella incidence in eight European countries: no correlation with incidence of reported cases. *BMC Public Health*.

[19] Helms M., Ethelberg S., Molbak K. (2005). International *Salmonella* Typhimurium DT104 infections, 1992–2001. *Emerging Infectious Diseases*.

[20] Carattoli A., Filetici E., Villa L., Dionisi A. M., Ricci A., Luzzi I. (2002). Antibiotic resistance genes and *Salmonella* genomic island 1 in *Salmonella enterica* serovar Typhimurium isolated in Italy. *Antimicrobial Agents and Chemotherapy*.

[21] Cawthorne A., Galetta P., Massari M., Dionisi A. M., Filetici E., Luzzi I. (2006). *Salmonella* typhimurium DT104, Italy. *Emerging Infectious Diseases*.

[22] Gatto A. J., Peters T. M., Green J. (2006). Distribution of molecular subtypes within Salmonella enterica serotype Enteritidis phage type 4 and S. Typhimurium definitive phage type 104 in nine European countries, 2000–2004: results of an international multi-centre study. *Epidemiology and Infection*.

[23] Dionisi A. M., Graziani C., Lucarelli C. (2009). Molecular characterization of multidrug-resistant strains of salmonella enterica serotype typhimurium and monophasic variant (S. 4,[5],12:i:-) isolated from human infections in Italy. *Foodborne Pathogens and Disease*.

[24] Targant H., Ponsin C., Brunet C. (2010). Characterization of resistance genes in multidrug-resistant *Salmonella enterica* serotype typhimurium isolated from diseased cattle in France (2002 to 2007). *Foodborne Pathogens and Disease*.

[25] Lawson A. J., Dassama M. U., Ward L. R., John Threlfall E. (2002). Multiply Resistant (MR) *Salmonella enterica* serotype Typhimurium DT 12 and DT 120: A case of MR DT 104 in disguise?. *Emerging Infectious Diseases*.

[26] Majtanova L., Majtan J., Majtan V. (2011). Trends in phage types of Salmonella enterica serovars Enteritidis and Typhimurium isolated in Slovakia from 1995 to 2009. *Diagnostic Microbiology and Infectious Disease*.

[27] Fantasia M., Filetici E., Arena S., Mariotti S. (1998). Serotype and phage type distribution of salmonellas from human and nonhuman sources in Italy in the period 1973–1995. *European Journal of Epidemiology*.

[28] Pasquali F., De Cesare A., Ricci A., Kehrenberg C., Schwarz S., Manfreda G. (2004). Phage types, ribotypes and tetracycline resistance genes of *Salmonella enterica* subsp. enterica serovar Typhimurium strains isolated from different origins in Italy. *Veterinary Microbiology*.

[29] Hopkins K. L., Kirchner M., Guerra B. (2010). Multiresistant Salmonella enterica serovar 4,[5],12:i:- in Europe: a new pandemic strain?. *Eurosurveillance*.

[30] EFSA (European Food Safety Authority) (2008). Report of the Task Force on Zoonoses Data Collection on the analysis of the baseline survey on the prevalence of *Salmonella* in slaughter pigs, Part A. *The EFSA Journal*.

[31] Ethelberg S., Wingstrand A., Jensen T. (2008). Large outbreaks of *Salmonella* typhimurium infection in Denmark in 2008. *Eurosurveillance*.

[32] Best E. L., Hampton M. D., Ethelberg S., Liebana E., Clifton-Hadley F. A., Threlfall E. J. (2009). Drug-resistant *Salmonella* Typhimurium DT 120: use of PFGE and MLVA in a putative international outbreak investigation. *Microbial Drug Resistance*.

[33] Mandilara G., Lambiri M., Polemis M., Passiotou M., Vatopoulos A. (2013). Phenotypic and molecular characterisation of multiresistant monophasic *Salmonella* typhimurium (1,4,[5],12:I:-) in Greece, 2006 to 2011. *Eurosurveillance*.

[34] Paranthaman K., Haroon S., Latif S. (2013). Emergence of a multidrug-resistant (ASSuTTm) strain of *Salmonella enterica* serovar typhimurium DT120 in England in 2011 and the use of multiple-locus variable-number tandem-repeat analysis in supporting outbreak investigations. *Foodborne Pathogens and Disease*.

[35] Bonardi S., Bassi L., Brindani F. (2013). Prevalence, characterization and antimicrobial susceptibility of *Salmonella enterica* and *Yersinia enterocolitica* in pigs at slaughter in Italy. *International Journal of Food Microbiology*.

